# Current practices in antibiotic prophylaxis for patients on complement inhibitors: a multicenter survey across ERN ERKnet centers

**DOI:** 10.1093/ckj/sfag282

**Published:** 2026-08-21

**Authors:** Savino Sciascia, Edoardo Terzolo, Roberta Fenoglio, Dario Roccatello, Stefanie Haeberle, Franz Schaefer, Nicole van de Kar, Marina Vivarelli, TMA ERN-ERKnet WG, Malgorzata Stanczyk, Stephanie Tellier, Marius Miglinas, Ana Teixeira, Rene Andersen, Huib De Jong, Josefina Santos Lascasas, Stathis Tsiakas, Adrian Lungu, Jerome Harambat, Caroline Duineveld, Laurent Mesnard, Luigi Cirillo, Enrique Morales, Giuseppe Grandaliano, Stéphane Decramer, Atif Awan, Ann Raes, Jakub Zieg, Mireya Carratala, Pedro Arango Sancho, Thomas Simon, Enrique Morales, Ann Raes, Carmen De Lucas Collantes, Nathalie Godefroid, Enrico Vidal, Alena Parikova, Johann Morelle, Cavero Teresa, Miquel Blasco, Evelien Snauwaert, Adrian Lungu, Alvaro Madrid, Martin Konrad

**Affiliations:** Department of Clinical and Biological Sciences, University Center of Excellence on Nephrologic, Rheumatologic and Rare Diseases (ERK-Net, ERN-Reconnect and RITA-ERN Member) with Nephrology and Dialysis Unit and Center of Immuno-Rheumatology and Rare Diseases (CMID), Coordinating Center of the Interregional Network for Rare Diseases of Piedmont and Aosta Valley, San Giovanni Bosco Hub Hospital ASL Città di Torino, Torino, Italy; Department of Clinical and Biological Sciences, University Center of Excellence on Nephrologic, Rheumatologic and Rare Diseases (ERK-Net, ERN-Reconnect and RITA-ERN Member) with Nephrology and Dialysis Unit and Center of Immuno-Rheumatology and Rare Diseases (CMID), Coordinating Center of the Interregional Network for Rare Diseases of Piedmont and Aosta Valley, San Giovanni Bosco Hub Hospital ASL Città di Torino, Torino, Italy; Department of Clinical and Biological Sciences, University Center of Excellence on Nephrologic, Rheumatologic and Rare Diseases (ERK-Net, ERN-Reconnect and RITA-ERN Member) with Nephrology and Dialysis Unit and Center of Immuno-Rheumatology and Rare Diseases (CMID), Coordinating Center of the Interregional Network for Rare Diseases of Piedmont and Aosta Valley, San Giovanni Bosco Hub Hospital ASL Città di Torino, Torino, Italy; Department of Clinical and Biological Sciences, University Center of Excellence on Nephrologic, Rheumatologic and Rare Diseases (ERK-Net, ERN-Reconnect and RITA-ERN Member) with Nephrology and Dialysis Unit and Center of Immuno-Rheumatology and Rare Diseases (CMID), Coordinating Center of the Interregional Network for Rare Diseases of Piedmont and Aosta Valley, San Giovanni Bosco Hub Hospital ASL Città di Torino, Torino, Italy; Division of Pediatric Nephrology, Department of Pediatrics, Faculty of Medicine, Heidelberg University, Heidelberg, Germany; Division of Pediatric Nephrology, Department of Pediatrics, Faculty of Medicine, Heidelberg University, Heidelberg, Germany; Department of Pediatric Nephrology, Amalia Children’s Hospital, Radboud University Medical Center, Nijmegen, The Netherlands; Laboratory of Nephrology and Clinical Trial Center, Bambino Gesù Children’s Hospital IRCCS, Rome, Italy

**Keywords:** antibiotic prophylaxis, complement inhibitors, eculizumab, ERKNet, pediatric nephrology, rare diseases, ravulizumab, transplant

## Abstract

**Background:**

Complement inhibitors such as eculizumab and ravulizumab are increasingly used to treat rare complement-mediated diseases. While effective, these therapies impair host defenses against encapsulated bacteria, leading to a heightened risk of invasive infections. Although vaccination is standard, the role and implementation of antibiotic prophylaxis remain inconsistent across clinical settings.

**Methods:**

A survey was conducted among European Reference Network for Rare Kidney Diseases (ERN ERKNet) centers to assess current practices regarding antibiotic prophylaxis in patients receiving complement-inhibition therapy. The survey addressed four clinical scenarios—pediatric vs. adult, transplant recipient vs. non-transplant—and explored three domains: indications for prophylaxis, choice of antibiotic, and duration.

**Results:**

Twenty-seven centers from 16 European countries participated. Systematic prophylaxis was widely endorsed, particularly for pediatric patients and transplant recipients. Penicillin-class antibiotics were preferred as first-line agents across all groups. In pediatric settings, phenoxymethylpenicillin and amoxicillin were most commonly used, whereas adult regimens included penicillin or fluoroquinolones for those with allergies. Most respondents recommended continuing prophylaxis throughout the duration of complement-inhibition therapy, regardless of vaccination status. The survey revealed areas of heterogeneity in practice, especially regarding allergy management and the duration of post-vaccination coverage.

**Conclusions:**

This ERKNet survey identifies prevailing practices and variation in antibiotic prophylaxis for patients receiving complement-inhibition therapy. Based on these findings, we propose consensus guidance to support harmonized, risk-adapted prophylaxis strategies across pediatric and adult populations. These guidance statements aim to address an important unmet need in infection prevention among complement-inhibited patients.

KEY LEARNING POINTS **What was known:**

- Complement inhibitors substantially increase susceptibility to invasive infections caused by encapsulated bacteria, particularly *Neisseria meningitidis*.
- Although vaccination is routinely recommended before complement inhibition, the indication, choice and duration of antibiotic prophylaxis remain incompletely defined.
- Clinical practice varies across countries and treatment settings, particularly for children, transplant recipients, vaccinated patients, and individuals with penicillin allergy.

**This study adds:**

- This survey provides the first multicenter overview of antibiotic prophylaxis practices across 27 ERKNet centers in 16 European countries.
- Most centers supported systematic prophylaxis, particularly in pediatric and transplant populations, with penicillin-class antibiotics preferred as first-line treatment.
- Most respondents favored continuing prophylaxis throughout complement-inhibitor treatment, although important variation remained regarding alternative agents, vaccination status, and discontinuation strategies.

**Potential impact:**

- These findings may support more consistent, risk-adapted prophylaxis protocols for patients receiving complement inhibitors across pediatric and adult nephrology services.
- The proposed ERKNet guidance may help clinicians standardize antibiotic selection, duration, and management of penicillin allergy while accounting for transplant status and individual infection risk.
- Greater harmonization could improve patient safety, reduce preventable invasive infections, and inform future international guidance, prospective studies, and antimicrobial-stewardship policies.

## INTRODUCTION

Complement inhibition has emerged as a cornerstone therapy for a growing number of rare and ultra-rare diseases, including atypical hemolytic uremic syndrome, paroxysmal nocturnal hemoglobinuria, and C3 glomerulopathy. Agents such as eculizumab and ravulizumab, which target the terminal complement component C5, offer potent control of complement-mediated pathology. However, these therapies are associated with an increased risk of infections, particularly by encapsulated organisms such as *Neisseria meningitidis*, due to their mechanism of action impairing terminal complement activity, which is essential for opsonophagocytic and bactericidal responses [1].

International guidelines universally recommend meningococcal vaccination prior to initiating complement inhibitors, and in some cases, also recommend vaccination against pneumococcus and *Haemophilus influenzae* type b [2–4]. However, the optimal strategy for antibiotic prophylaxis in these patients—particularly regarding its indication, choice of agent, and duration—remains insufficiently defined. This issue is especially complex in pediatric populations, in immunocompromised patients such as transplant recipients, and in cases where vaccination is incomplete or contraindicated.

In clinical practice, the implementation of antibiotic prophylaxis varies significantly across centers and countries, reflecting both local epidemiology and differing interpretations of residual infection risk. While some institutions adopt a systematic approach to prophylaxis in all patients receiving anti-complement therapy, others reserve it for specific clinical scenarios (i.e. children <5 years of age, young adults living in communities such as college, immunocompromised patients, and transplant recipients on immunosuppression) or for defined time periods (usually 14 days) following vaccination.

To characterize current clinical practices across Europe, we conducted a structured survey within the European Rare Kidney Disease Reference Network (ERN ERKNet). The survey aimed to explore the indications, preferred antibiotic agents, and recommended duration of prophylaxis in adult and pediatric patients, both in general and in the context of solid organ transplantation. By mapping existing heterogeneity and identifying consensus areas, this study aims to inform future guidelines and harmonize infection prevention strategies in patients undergoing complement inhibition therapy.

## MATERIALS AND METHODS

### Study design and objective

We conducted a survey within the ERN ERKNet to assess current practices regarding antibiotic prophylaxis in patients receiving anti-complement therapy. The objective was to characterize institutional and clinician-level decision-making across three key domains: (i) indication for antibiotic prophylaxis, (ii) choice of antibiotic agent, and (iii) recommended duration of prophylaxis. The survey was developed under the scientific supervision of ERKnet-TMA WG, in consultation with experts in rare kidney diseases and infectious disease prevention.

### Survey development

The questionnaire was structured around four clinical scenarios to reflect real-world decision-making contexts:

- **Pediatric patients** receiving anti-complement therapy.
- **Adult patients** receiving anti-complement therapy.
- **Pediatric transplant recipients** receiving anti-complement therapy.
- **Adult transplant recipients** receiving anti-complement therapy.

Each scenario was assessed across the three predefined domains:

- **Indication:** clinical criteria for initiating antibiotic prophylaxis.
- **Antibiotic choice:** preferred agents, alternatives in case of allergy, and contraindications.
- **Duration:** timing in relation to vaccination and continuation throughout complement inhibition therapy.

The survey also included items on non-antibiotic preventive measures, institutional protocols, and open-ended questions to collect qualitative insights and comments.

Following the initial analysis of survey responses, the TMA ERN-ERKnet Working Group (WG) convened to establish clinical guidance. A two-stage iterative process was employed: first, survey data were categorized by the level of agreement (e.g. universal, majority, or heterogeneous). Second, draft recommendations were circulated among core WG members, and specific guidance was finalized only after achieving a supermajority agreement (>75%) on the proposed statements. This approach allowed for the synthesis of empirical survey data with expert clinical interpretation to address areas where practice heterogeneity was high.

### Data collection and participants

The survey was distributed electronically in October 2024 to all ERKNet-affiliated centers managing patients with rare kidney diseases, with a focus on pediatric and adult nephrologists, immunologists, and transplant specialists. Responses were collected using a secure online platform. Participation was voluntary, and one response per center was encouraged to capture institutional rather than individual-level practices.

Six members of the ERKNet working group contributed to data extraction and interpretation of the survey findings. Draft practical statements were formulated on the basis of the survey results and the available literature. These statements were subsequently discussed, revised, and finalized during an in-person meeting held in conjunction with the annual ERKNet meeting. Consensus was achieved through discussion among the participating experts.

### Data analysis

Survey responses were exported into a structured dataset. Descriptive statistics were used to summarize frequencies of responses per question and clinical scenario. For open-ended questions, thematic analysis was performed to identify recurring patterns and divergent views. Data were stratified by patient age group and transplant status to explore differences in practice across scenarios.

## RESULTS

### Respondent characteristics

The questionnaire was circulated to all 72 ERKNet expert centers. Thirty-nine clinicians completed the survey, including 25 pediatric nephrologists or pediatricians and 14 adult nephrologists or internal medicine physicians. After consolidating multiple responses from the same institution, 27 uniquely identifiable ERKNet-affiliated centers were represented, corresponding to a center-level response rate of 37.5% (27/72). Response completeness and the relevant denominator for each survey item are reported in Supplementary Table 1S.

### Pediatric setting

Systematic antibiotic prophylaxis was endorsed by 67% of the participating centers, regardless of vaccination status, especially in children who were transplant recipients or receiving immunosuppressive therapies (Table 1). Free-text comments revealed that many centers applied this approach universally to pediatric patients, citing the high residual risk of invasive *N. meningitidis* infections.

**Table 1:** Summary of the consensus on *the use of* Antibiotic prophylaxis in pediatric and adult patients receiving anti-complement therapies.

*Guidance of the use of* Antibiotic prophylaxis in pediatric patients receiving anti-complement therapies
A consensus has been obtained on the use of antibiotic prophylaxis for pediatric patients receiving anti-complement therapy.
If not vaccinated, prophylaxis for pediatric patients receiving anti-complement therapy is supported by a general consensus.
If vaccinated, prophylaxis for at least 2 weeks since vaccination (completed schedule) is supported by a general consensus.
Due to the residual risk of infection, many centers establish that antibiotic prophylaxis should be continued independently of vaccination status in the pediatric patient on complement-inhibition therapy, especially if transplant-recipient.
The use of penicillin as the first-choice agent in prophylaxis for pediatric patients, including transplant-recipient, is supported by a general consensus.
If a penicillin allergy is documented, a case-by-case analysis of risk-benefit ratio is warranted.
***Guidance of the use of* Antibiotic prophylaxis in adults receiving anti-complement therapies**
A consensus has been obtained on the use of antibiotic prophylaxis for adult patients receiving anti-complement therapy.
Antibiotic prophylaxis for transplant-recipient adult patients and for non-vaccinated adult patients receiving complement-inhibition therapy is systematically suggested.
The use of penicillin as the first-choice agent in prophylaxis for the adult patient, including adult transplant-recipient, is supported by a general consensus.
If a penicillin allergy is documented, a fluoroquinolone may represent a II-line choice.
Even if consensus was not achieved, the majority of the centers supports that duration of antibiotic prophylaxis should be extended at least for the entire duration of complement-inhibition therapy due to the residual risk of infection for adult patients, especially if transplant-recipients.
Additional consideration:
Any potential benefit of prolonged antibiotic prophylaxis should be balanced against the risks of adverse effects, clinically relevant drug interactions, reduced long-term adherence, and antimicrobial resistance.

Phenoxymethylpenicillin was the most frequently recommended agent in children (Table 2). Amoxicillin was also commonly used, particularly in infants. Cephalosporins appear to be an uncommon approach for prophylaxis. In the case of penicillin allergy, macrolides such as azithromycin were the most employed class of antibiotics, although several respondents raised concerns about QT prolongation and drug interactions, particularly in children receiving calcineurin inhibitors.

**Table 2:** From consensus to guidance: suggested antibiotic regimens.

Antibiotic regimen	Pros	Cons
Penicillins (e.g. Penicillin V 500 mg bid—for children ≥3 years of age 250 mg orally twice daily; for children <3 years of age 125 mg orally twice daily) [8]	Overall good tolerability profile	Higher rates of allergy Intermediate/resistant strains rising [9]
Fluoroquinolones (e.g. ciprofloxacin 500 mg/day–10-20 mg/kg in pediatric patients)	Valid alternative if *N. meningitidis* resistance is low	Toxicity increases with higher cumulative dose—better for shorter courses Restrict use in pediatric patients [10]
Macrolides (e.g. Azithromycin 500 mg/day—5 mg/kg/day for pediatric patients)	Suitable for pediatric and pregnancy Good sensitivity spectrum *in vitro* [9]	Consider cardiac toxicity and hepatotoxicity (comorbid patients and interfering drugs) [11] With prolonged course with Azithromycin consider ototoxicity [12]

Consider cumulative dose and exposure time when choosing different regimens of antibiotic prophylaxis.

Regarding duration, most centers recommended maintaining prophylaxis for the entire course of complement inhibitor therapy. Some added that prophylaxis should continue for at least 2–3 weeks after the final dose of meningococcal vaccine, especially in younger or unvaccinated children. Only a minority of responses proposed conditional prophylaxis strategies (e.g. depending on vaccination status or complement assay evaluation).

### Adult setting

While adult-specific responses were less systematically detailed, several key patterns emerged. Among centers that addressed adult patients, most indicated a preference for continued prophylaxis throughout the duration of complement-inhibition therapy. As in the pediatric setting, phenoxymethylpenicillin 500 mg once or twice daily or **amoxicillin** were commonly prescribed. In cases of penicillin allergy, other categories (Table 2) were generally preferred, reflecting clinician familiarity and a broader safety profile in adult populations.

The rationale for prolonged prophylaxis mirrored that used in children: ongoing susceptibility to encapsulated organisms despite full vaccination and the lack of robust clinical data on protective antibody titers in complement-deficient states. A few responses alluded to potential tailoring of prophylaxis based on complement recovery (e.g. CH50 levels), but these approaches were rare.

### From survey to guidance

The marked heterogeneity in practices observed across ERKNet centers—particularly regarding the choice and duration of antibiotic prophylaxis in patients receiving complement-inhibition therapy—highlighted the need for harmonized clinical guidance. While most respondents supported systematic prophylaxis, especially in pediatric and transplant settings, considerable variation was noted in the specific antibiotic agents used, the approach to penicillin allergy, and the timing of discontinuation in relation to vaccination status. Based on this empirical landscape and expert consensus, we formulated pragmatic guidance aiming to ensure consistent and safe preventive strategies across rare disease networks. These statements, derived from collective clinical expertise and grounded in the prevailing practices identified through our survey, provide a framework for standardized prophylaxis tailored to patient age, immune status, and treatment context​

## DISCUSSION

This survey of ERKNet-affiliated centers provides the first systematic overview of current practices surrounding antibiotic prophylaxis in patients receiving complement-inhibition therapy across Europe. Despite the recognized infectious risks associated with anti-complement agents, particularly against *N. meningitidis*, there has been a lack of harmonized guidance regarding prophylactic antibiotic strategies. Our findings confirm that the majority of centers recommend antibiotic prophylaxis systematically, particularly in pediatric patients and transplant recipients, yet important variation persists regarding agent selection, duration, and conditional use based on vaccination status.

The most consistent signal across responses was the endorsement of continuous prophylaxis throughout the duration of complement-inhibition therapy. This aligns with the known mechanism by which terminal complement blockade impairs host defense, particularly against encapsulated organisms such as *N. meningitidis, Streptococcus pneumoniae*, and *H. influenzae* type b [5, 6]. Several respondents extended this precautionary approach to all patient groups, regardless of age, transplant status, or vaccination history. This reflects a widely shared perception of residual infection risk, despite current immunization protocols, and is consistent with post-marketing surveillance data showing breakthrough infections even in fully vaccinated individuals [1].

Penicillin-class antibiotics, particularly phenoxymethylpenicillin and amoxicillin, were widely preferred as first-line agents, especially in pediatric patients. In adults, similar preferences were expressed, albeit with more frequent use of fluoroquinolones as a second-line option in those with penicillin allergy. Notably, responses also highlighted practical concerns about macrolides in immunosuppressed patients, including QT prolongation and interactions with calcineurin inhibitors [7]. This underlines the need for specific antimicrobial stewardship strategies in this patient population, particularly among transplant recipients.

Our findings informed the development of practical recommendations, which are stratified by age group and transplant status. These include the systematic use of penicillin-based prophylaxis, individualized consideration in cases of allergy, and extension of prophylaxis through the duration of therapy, regardless of vaccination status. This framework aims to support clinicians in making consistent, evidence-informed decisions and provides a foundation for future prospective validation.

This study has several strengths. It captures real-world practices from a diverse network of expert centers and addresses a pressing unmet need in the management of rare kidney disease patients treated with anti-complement agents. However, it also has limitations. The survey design relied partly on free-text responses, which led to variability in the depth and clarity of answers. In addition, the sample size, while appropriate for a rare disease context, limits formal subgroup analysis. Finally, the recommendations derived from this work reflect expert consensus in the absence of randomized trials and should be interpreted accordingly.

In conclusion, this survey highlights both the areas of convergence and divergence in antibiotic prophylaxis practices for patients receiving complement-inhibition therapy. Based on these findings and expert interpretation, we propose a pragmatic set of guidance statements to support harmonized, safe, and effective infection prevention strategies across the ERKNet network and beyond.

## Supplementary Material

sfag282_Supplemental_File

## Data Availability

The data underlying this article will be shared on reasonable request to the corresponding author.
