# Supplementary material for: Current practices in antibiotic prophylaxis for patients on complement inhibitors: a multicenter survey across ERN ERKnet centers
Source: Clin Kidney J. 2026 Aug 21;19(9):sfag282. doi: 10.1093/ckj/sfag282 (PMC13548869; doi:10.1093/ckj/sfag282)
Supplement: Supplementary file 1 [file sfag282_supplemental_file.zip › Supplementary Material 1.docx]

Supplementary Material 1

Table 1S: Response completeness and the relevant denominator for each survey item

| **Survey item** | **Eligible respondents, n** | **Evaluable complete responses, n (%)** | **Paritial responses, n (%)** |
| --- | --- | --- | --- |
| Respondent practice: pediatric versus adult | 39 | 39 (100%) | 0 (0%) |
| Particular clinical scenarios in which antibiotic prophylaxis is prescribed | 39 | 38 (97.4%) | 1 (2.6%) |
| Routine antibiotic prophylaxis in pediatric patients receiving complement inhibitors | 25 | 24 (96.0%) | 1 (4.0%) |
| Routine antibiotic prophylaxis in pediatric transplant recipients receiving complement inhibitors | 25 | 22 (88.0%) | 3 (12.0%) |
| Routine antibiotic prophylaxis in adult patients receiving complement inhibitors | 14 | 12 (85.7%) | 2 (14.3%) |
| Routine antibiotic prophylaxis in adult transplant recipients receiving complement inhibitors | 14 | 12 (85.7%) | 2 (14.3%) |

The survey included 39 clinicians: 25 pediatric nephrologists or pediatricians and 14 adult nephrologists or internal medicine physicians. Pediatric-specific questions were analyzed only among pediatric respondents, and adult-specific questions only among adult respondents.
