# Supplementary material for: Current practices in antibiotic prophylaxis for patients on complement inhibitors: a multicenter survey across ERN ERKnet centers
Source: Clin Kidney J. 2026 Aug 21;19(9):sfag282. doi: 10.1093/ckj/sfag282 (PMC13548869; doi:10.1093/ckj/sfag282)
Supplement: Supplementary file 1 [file sfag282_supplemental_file.zip › Survey.pdf]

Antibiotic Prophylaxis in Patients Receiving Anti-Complement Therapy

Dear members of ERKNet TMA WG,
We are writing to invite you to participate in an important Delphi survey conducted by ERKNet, aimed at consolidating expert opinions on the management of antibiotic prophylaxis in patients receiving anti-complement therapy. This survey is part of an ongoing effort to standardize care practices and improve patient outcomes across various medical settings.

About the Survey:
This Delphi survey seeks to gather insights from leading healthcare professionals like yourself on the preferred strategies for antibiotic prophylaxis across different patient demographics, including adults, pediatrics, and transplanted patients both in adult and pediatric categories. The survey addresses scenarios where antibiotic prophylaxis might be recommended and explores alternatives for patients with specific allergies.

Why Your Participation Matters:
Your expertise and responses will be instrumental in developing a comprehensive understanding of current practices and emerging consensus in this specialized area of healthcare. The aggregate findings will contribute to a peer-reviewed publication and may inform clinical guidelines, impacting patient care globally.

Delphi Process:

The Delphi method is a structured communication technique, originally developed as a systematic, interactive forecasting method which relies on a panel of experts. You will be asked to complete a questionnaire in two rounds. After each round, a summary of the experts' forecasts from the previous round is provided, and the experts are encouraged to revise their earlier answers in light of the replies of other members of their panel. It is believed that during this process the range of the answers will decrease, and the group will converge towards the "correct" answer.

First name, last name:

XX

! This question requires an answer.

* **Email address:**

XX

Institution:

- ◯ Aachen, University Hospital
- ◯ Aarhus, University Hospital

- ◯ Amsterdam, Amsterdam, Academic University Medical Center (AUMC)
- ◯ Athens, Laiko General Hospital of Athens
- ◯ Barcelona, Hospital Clinic de Barcelona
- ◯ Barcelona, Hospital de la Santa Creu I Sant Pau and Fundacio Puigvert/IUNA
- ◯ Barcelona, Hospital San Joan de Deu
- ◯ Barcelona, Hospital Universitari Vall d'Hebron
- ◯ Bari, AOU Consorziale policlinico di Bari-Ospedale Pediatrico Giovanni XXIII
- ◯ Bergamo, Azienda Ospedaliera Papa Giovanni XXIII
- ◯ Berlin, Charité - Universitätsmedizin Berlin
- ◯ Bologna, AOU Policlinico S. Orsola Bologna
- ◯ Bordeaux, University Hospital
- ◯ Brescia, ASST Spedali Civili Brescia
- ◯ Brussels, Cliniques universitaires Saint-Luc - UCL
- ◯ Bucharest, Fundeni Clinical Institute
- ◯ Budapest, Semmelweis University

◯ Cologne, University Hospital

◯ Copenhagen, Rigshospitalet University Hospital

◯ Cracow, University Children's Hospital of Cracow

◯ Dublin, Beaumont Hospital

◯ Dublin, Children`s Health Ireland (CHI) at Temple Street

◯ Erlangen, University Hospital

◯ Essen, University Children`s Hospital

◯ Florence, Meyer Children’s Hospital IRCCS

◯ Gdansk, University Clinical Centre Gdansk

◯ Genoa, Istituto Giannina Gaslini

◯ Ghent, UZ Ghent

◯ Groningen, University Medical Center

◯ Hamburg, University Medical Center Hamburg-Eppendorf

◯ Hannover, Hannover Medical School

◯ Heidelberg, University Hospital

- ◯ Helsinki, Children´s Hospital, Helsinki University Hospital
- ◯ Innsbruck, Medical University Innsbruck
- ◯ Leiden, Leiden University Medical Center
- ◯ Leipzig, University Hospital
- ◯ Leuven, University Hospitals Leuven
- ◯ Limoges, University Hospital
- ◯ Ljubljana, University Medical Centre
- ◯ Lodz, Instytut
- ◯ Luxembourg, Centre Hospitalier de Luxembourg
- ◯ Lyon, Hôpital Femme Mère Enfant & Université de Lyon
- ◯ Madrid, Hospital Universitario 12 de Octubre
- ◯ Milan, IRCCS Ca' Granda Ospedale Maggiore Policlinico
- ◯ Montpellier, University Hospital
- ◯ Msida, Mater Dei Hospital
- ◯ Münster, University Hospital

- ◯ Naples, A.O. Santobono-Pausilipon
- ◯ Naples, UOC Nephrology and Dialysis - Università della Campania
- ◯ Nijmegen, Radboud UMC
- ◯ Odense, Odense University Hospital
- ◯ Oslo, University Hospital and Institute of Clinical Medicine
- ◯ Padua, Azienda Ospedaliera di Padova
- ◯ Paris, APHP Hôpital Henri Mondor, Paris-Est Creteil
- ◯ Paris, Hopital Europeen Georges Pompidou
- ◯ Paris, Hospital Robert Debre
- ◯ Paris, Necker-Enfants Malades University Hospital
- ◯ Paris, Tenon Hospital, Assistance Publique Hôpitaux de Paris
- ◯ Porto, Centro Hospitalar Universitário do Porto
- ◯ Prague, Institute for Clinical and Experimental Medicine IKEM Prague
- ◯ Prague, University Hospital Motol
- ◯ Riga, Pauls Stradins University Hospital

- ◯ Riga, University Children Hospital
- ◯ Rome, Gemelli Hospital
- ◯ Rome, Rome, Bambino Gesù Children's Hospital
- ◯ Rosenheim, RoMed Klinikum Rosenheim
- ◯ Rotterdam, Erasmus MC: University Medical Center Rotterdam
- ◯ Sevilla, Complejo Hospitalario Regional Virgen del Rocio
- ◯ Siena, University Hospital
- ◯ Stockholm, Karolinska University Hospital
- ◯ Tallinn, North Estonian Medical Center
- ◯ Tartu, Tartu University Hospital
- ◯ Toulouse, Toulouse, University Hospital
- ◯ Turin, AOU Città della Salute e della Scienza di Torino - Molinette Hospital
- ◯ Turin, AOU CSS di Torino - Regina Margherita Hospital
- ◯ Turin, University of Torino-Ospedale HUB Torino Nord
- ◯ Utrecht, University Medical Centre

◯ Vienna, Department of Pediatrics; Vienna General Hospital

◯ Vilnius, Vilnius University Hospital Santaros Klinikos

Country:

XXX

Next

Antibiotic Prophylaxis in Patients Receiving Anti-Complement Therapy

1. Antibiotic prophylaxis in patients receiving anti-complement therapy

1.1 Do you consider any particular scenarios for prescribing antibiotic prophylaxis in patients receiving anti-complement therapy?

- ◯ Yes
- ◯ No

1.1.1 If yes, please select (more answers allowed):

- ☐ immunosuppression due to other therapies
- ☐ living in a community
- ☐ age (please specify in the comment box below)
- ☐ type of anti-complement agent (please specify in the comment. box below)

☐ Other (please specify)

* **Who is answering the survey?**

◯ Pediatric nephrologist/ pediatrician

◯ Adult nephrologist/ internal medicine physician

Prev Next

Antibiotic Prophylaxis in Patients Receiving Anti-Complement Therapy

2. Pediatrics

2.1 Do you systematically recommend antibiotic prophylaxis for pediatric patients receiving anti-complement therapy?

◯ Yes

◯ No

2.2 Preferred antibiotic prophylaxis agent and dosage in pediatric patients receiving anti-complement therapy

2.3 Preferred antibiotic prophylaxis scheme duration in pediatric patients receiving

anti-complement therapy

2.4 How do you approach antibiotic prophylaxis in pediatric patients with a penicillin allergy receving anti-complement therapy?

Alternative antibotics

Non-antibiotic prophylactic measures

No prophylaxis

3. Pediatric transplanted

3.1 Do you systematically recommend antibiotic prophylaxis for pediatric transplanted patients receiving anti-complement therapy?

- ◯ Yes
- ◯ No

3.2 Preferred antibiotic prophylaxis agent and dosage in pediatric transplanted receiving anti-complement therapy

3.3 Preferred antibiotic prophylaxis scheme duration in pediatric tranplanted receiving anti-complement therapy

3.4. How do you approach antibiotic prophylaxis in pediatric transplanted with a penicillin allergy receiving anti-complement therapy?

Alternative antibotics

Non-antibiotic prophylactic measures

No prophylaxis

Prev Next

Antibiotic Prophylaxis in Patients Receiving Anti-Complement Therapy

4. Adults

4.1 Do you systematically recommend antibiotic prophylaxis for adult patients receving anti-complement therapy?

- ◯ Yes
- ◯ No

4.2 Preferred antibiotic prophylaxis agent and dosage in adults receving anti-complement therapy

4.3 Preferred antibiotic prophylaxis scheme duration in adults receiving anti-complement therapy

4.4 How do you approach antibiotic prophylaxis in patients with a penicillin allergy receiving anti-complement therapy?

Alternative antibiotics

Non-antibiotic prophylactic measures

No prophylaxis

4.5 Do you systematically recommend antibiotic prophylaxis for transplanted adult patients receiving anti-complement therapy?

◯ Yes

◯ No

5. Adult Transplantation

5.1 Do you systematically recommend antibiotic prophylaxis for transplanted adult patients receiving anti-complement therapy?

- ◯ Yes
- ◯ No

5.2 Preferred antibiotic prophylaxis agent and dosage in transplanted adults receiving anti-complement therapy

5.3 Preferred antibiotic prophylaxis scheme duration in transplanted adults receiving anti-complement therapy

5.4 How do you approach antibiotic prophylaxis in transplanted patients with a penicillin allergy receiving anti-complement therapy?

Alternative antibiotics	
Non-antibiotic prophylactic measures	
No prophylaxis	

Prev Next
